# Schirrous invasive ductal carcinoma of the breast overexpress p53 oncoprotein

**DOI:** 10.1590/S1516-31802001000100002

**Published:** 2001-01-02

**Authors:** Flávia Silva Ferrini, Marcos Antonio Rossi, Mário Mourão, Fernando Augusto Soares

**Keywords:** Breast neoplasms, p53 oncoprotein, c-erbB-2 oncoprotein, Hormonal receptors, Invasive ductal carcinoma of the breast, Neoplasias de mama, Oncoproteína p53, Oncoproteína c-erbB-2, Receptores hormonais, Carcinoma ductal infiltrante da mama

## Abstract

**CONTEXT:**

Breast cancer is the most important neoplasm in adult women, and its worldwide incidence is growing. The tumoral stroma is very important for modulating the growth and invasion of the tumor itself. The relationship between these two components is not completely understood. Schirrous carcinoma is a variant of ductal invasive carcinoma in which the stroma is very desmoplastic, and the importance of this finding still a motive for debate in the literature.

**OBJECTIVE:**

To compare the desmoplastic reactions against biological markers, such as estrogen and progesterone receptors, oncoprotein c-erbB-2 and oncoprotein p53, with the objective of studying the relationship between the tumoral stroma and epithelial cancer cells.

**TYPE OF STUDY:**

Retrospective study.

**SETTING:**

Cancer Hospital A C Camargo and Faculty of Medicine of Ribeirão Preto, University of São Paulo, São Paulo, Brazil.

**SAMPLE:**

107 adult women operated because of ductal invasive carcinoma. The cases were separated into 4 groups according to the desmoplastic reaction – less than 15%, between 15-50%, 51-85%, and more than 85% fibrosis. The grade of fibrosis was determined by picrus-sirius staining and quantified by using a microscope with a stereo-imaging grid. Immunohistochemical methods were used to determine the expression of the hormonal receptors and c-erbB-2/p53 oncoprotein.

**MAIN MEASUREMENTS:**

Extent of desmoplastic reaction versus expression of estrogen and progesterone receptors, oncoprotein c-erbB-2, and oncoprotein p53.

**RESULTS:**

The results showed that schirrous carcinoma expresses oncoprotein p53 more frequently than other carcinomas with less extensive desmoplastic reaction. There were no differences between the grade of fibrosis and the other biological markers.

**CONCLUSION:**

The intense stromatous reaction in invasive ductal carcinoma may modulate the expression of p53. Further investigations should be made with the aim of understanding how this expression determines the proliferative activity in schirrous carcinoma, and whether this overexpression is secondary to mutation of the p53 gene or due to modulation of other molecules of the stroma.

## RESUMO

**CONTEXTO:**

Carcinoma da mama é a neoplasia maligna mais importante nas mulheres adultas e sua incidência apresenta crescimento em todo o mundo. O estroma tumoral é um compartimento morfológico muito importante na modulação do crescimento e invasão tumorais. As relações entre as células epiteliais e o respectivo estroma não são ainda completamente entendidas. Carcinoma esquirroso é uma variante do carcinoma ductal infiltrante usual da mama, caracterizado pelo extenso componente desmoplásico, e sua importância como tipo histológico especial é ainda motivo de debate na literatura.

**OBJETIVO:**

Comparar o grau de reação desmoplásica com a expressão de marcadores biológicos como os receptores de estrógeno e progesterona, oncoproteínas c-erbB-2 e p53, com o objetivo de estudar as inter-relações entre o estroma tumoral e as células epiteliais neoplásicas.

**TIPO DE ESTUDO:**

Estudo retrospectivo.

**LOCAL:**

Hospital do Câncer A C Camargo e Faculdade de Medicina de Ribeirão Preto, Universidade de São Paulo, São Paulo, Brasil.

**AMOSTRA:**

107 casos de mulheres adultas operadas do carcinoma ductal infiltrante da mama. Os casos foram separados em quatro grupos de acordo com a reação desmoplásica: grupo 1 – menos do que 15% do tumor era constituído por estroma; grupo 2 – estroma entre 15-50% de estroma; grupo 3 – entre 50-85% de fibrose; e grupo 4, com mais de 85% de fibrose. O grau de fibrose foi determinado utilizando-se a coloração de picrus-sirius vermelho e quantificado com ocular estequiométrica. Reações imuno-histoquímicas foram utilizadas para determinar a expressão dos receptores de estrógeno e progesterona e as oncoproteínas cerbB-2 e p53.

**VARIÁVEIS ESTUDADAS:**

Grau de reação desmoplásica versus expressão imunohistoquímica de receptores de estrógeno, progesterona e oncoproteínas c-erbB-2 e p53.

**RESULTADOS:**

Os resultados mostraram que os carcinomas esquirrosos superexpressam oncoproteína p53 mais freqüentemente do que todos os demais grupos de menor reação desmoplásica. Não houve diferenças com os demais marcadores biológicos

**CONCLUSÃO:**

A reação estromal desmoplásica pode ser reguladora da expressão da proteína p53 em carcinomas ductais infiltrantes da mama. Investigações futuras devem ser conduzidas para determinar como essa expressão diferenciada pode influir no índice proliferativo, e se esta superexpressão é decorrente da mutação de gene p53 ou devido a modulação por outros fatores embebidos no estroma neoplásico.

## INTRODUCTION

Breast cancer is the most common malignancy among women almost throughout the whole world, and its incidence has been growing over recent decades. According to the Brazilian National Institute of Cancer (INCA), 30,000 new cases/year are documented in Brazil, and more than 7000 deaths per year.^1^ Infiltrating ductal carcinoma (DC) is the most common form of breast cancer. Desmoplastic reaction is characteristic of DC, and the intensity of this reaction can be different from case to case. The interactions between the tumoral stroma and the neoplastic cells are very important, and the tumor stroma can act as a regulator of neoplastic behavior.^2^

The most important prognostic factors in DC are the clinical stage and the histological differentiation. Many other factors have been described as important in determining the biological behavior of DC cases. Morphological aspects, such as necrosis, nuclear grade, histo-logical grade, inflammatory response, mitosis index, vascular and perineural invasion, angio-genesis, as well biological markers like hormone receptors, c-erbB-2 oncoprotein, cathepsin, p53 protein, proliferative index, and cyclin D1, have been extensively reviewed in the literature.^3^

The capacity of the DC to cause a desmo-plastic reaction is well known, and its value as a prognostic factor has been debated in the literature. Recently, Hasebe et al. have shown that tumors with fibrotic foci have worse behavior than DC without fibrotic reaction.^4,5^ They have suggested that the presence of a fibrotic focus is an important predictor of early recurrence or death in patients with DC.^4^ Also, Cardone et al. have described a short survival time in patients with schirrous carcinoma, and a positive correlation between desmoplastic reaction and lymph node metastasis.^6^

The present study addresses the question of the relationship between the desmoplastic reaction and some of the most important biological markers in DC, such as hormone receptors, expression of c-erbB-2 and p53 oncoproteins.

## METHODS

One hundred and seven consecutive cases of invasive ductal carcinoma diagnosed in 1997 were retrieved from the files of the Department of Anatomical Pathology of the Cancer Hospital A C Camargo. All of the pathology specimens from the breast tumor were obtained by partial or radical mastectomy. All surgical specimens had been fixed in buffered formalin overnight. For histological evaluation, tissue sections were stained with hematoxylin and eosin. For evaluation of the desmoplastic reaction of the stroma, histochemical studies were performed with picrus-sirius hematoxylin staining.

Immunohistochemical studies were performed on formalin-fixed, paraffin-embedded tissue sections using the avidin-steptavidin-peroxidase method. To enhance the immunostaining, sections were mounted on silane-treated slides, baked at 56^°^C for 60 minutes, deparaffinized in xylene, rehydrated in graded alcohols, and rinsed. For heat-induced epitope retrieval, sections were placed in 0.01M citrate buffer at pH 6.0, and heated in a microwave oven for six cycles of 5 minutes each, followed by 20 minutes cooling at room temperature. Endogenous peroxidase activity was blocked using hydrogen peroxidase/methanol. This was followed by overnight incubation with the monoclonal antibodies for estrogen receptor (1D5, DAKO, dilution 1/50), progesterone receptor (1A6, DAKO, dilution 1/30), c-erbB-2 oncoprotein (c-erbB-2, DAKO, dilution 1/500), and p53 protein (DO-7, DAKO, dilution 1/100).

The specificity of the immunoreaction was verified by the use of known negative and positive controls. For the hormonal receptors we used residual normal mammary ducts as internal controls for the reaction. 3,3'diaminobenzine (Sigma) was used as a chromogen. Cases were interpreted as hormonal receptor positive or p53 protein positive if more than 10% of the neoplastic cells showed positive nuclear staining, and c-erbB-2 oncoprotein positive when a membrane pattern stain was shown in more than 10% of the cells.

To evaluate the desmoplastic reaction, the slides were stained with picrus-sirius red histo-chemical stain. The amount of fibrosis was evaluated using a stereo-imaging grid of 25 points mounted in the microscope eyepiece. We counted each point of the grid as either stromatous or epithelial. A total of 20 high-power fields (a total of 500 points) were counted and the result was expressed as the percentage of fibrosis in relation to epithelial areas. The results were divided into four groups: minimal desmoplastic reaction (less than 15% fibrosis), discrete desmoplastic reaction (between 15 and 50% fibrosis), prominent desmoplastic reaction (between 51 and 85% fibrosis), and schirrous carcinoma (more than 85% fibrosis).

### Statistical methods

Statistical analysis was performed using Graph Pad Prism v.2 software (San Diego, CA). We used the chi-squared test to compare the four groups. If the chi-squared test showed a significant result, we used Fisher's exact test to compare the groups. *P* values of less than 5% were judged to be statistically significant.

## RESULTS

The complete results are showed in the Table. The 107 cases of invasive ductal carcinoma of the breast were divided, in accordance with the desmoplastic reaction, into minimal fibrosis in 19.6% of the cases (21/107), discrete fibrosis in 37.4% (40/107), prominent fibrosis in 27.1% (29/107), and schirrous carcinoma in 15.9% (17/107).

**Table:** Expression of the biological markers in relation to the desmoplastic reaction pattern^a^.

Desmoplastic Reaction	Number of cases	Positive Estrogen receptor^b^	Positive Progesterone receptor^b^	Positive c-erbB-2 oncoprotein^b^	Positive p53 protein*
Minimal	21	10 (47.6%)	4 (19%)	12 (57.1%)	2 (9.5%)
Discrete	40	18 (45%)	10 (25%)	24 (60%)	9 (22.5%)
Prominent	29	16 (55.2%)	5 (17.2%	19 (65.5%)	10 (34.5%)
Schirrous carcinoma	17	53 (49.5%)	4 (23.5%)	12 (70.65)	12 (70.6%)
**Total**	**107**	**53 (49.5)**	**23 (24.6%)**	**40 (37.4%)**	**33 (30.8%)**

a *Results expressed as number of positive cases in each group;*

b *Non-significant result with P-value > 0.05;*

* *Significant result with P = 0.0003*

The estrogen and progesterone receptors and the c-erbB-2 oncoprotein did not shown any relation with the grade of desmoplastic reaction. Differently, p53 protein was significantly more expressed by the schirrous carcinoma cases in comparison with all other groups, and individually with each one of them.

## DISCUSSION

The main finding from this study is the overexpression of the p53 protein in cases of schirrous carcinoma in comparison with all other groups with relatively less fibrous stroma. We did not observed any other correlation between the grade of desmoplastic reaction and the expression of hormonal receptors and c-erbB-2 oncoprotein.

The p53 gene is located on the small arm of chromosome 17, and its protein product is present in virtually all normal tissues. The p53 gene is deleted or mutated in up to half of all human cancers, and seem to be the most common genetic change in human cancer.^7^ In normal cells, p53 is a negative regulator of cell division. When activated, the p53 protein stimulates the transcription of a gene encoding a Cdk inhibitor protein called p21, and this protein blocks the cell division cycle. The p53 protein has a very short life, and in normal concentrations is not detectable by immunohistochemical stains. Nuclear accumulation of p53 protein, as measured by immunohistochemistry, results from stabilization of the protein by nonsense mutations or interactions with other molecules of the stroma that determine an increase in its average life. Further studies to determine whether the overexpression of p53 proteins in cases of schirrous carcinoma is primary to genetic alterations of the p53 gene or modulated by the stroma will be the next step in understanding the result of this study better.

The relevance of the stroma in epithelial cell growth in normal development, as well as in breast cancer, has been demonstrated by various authors.^8-9^ The amount of thrombospondin, a potent stimulator of angiogenesis, in DC is significantly increased in comparison with what is observed in normal and benign tissues, and especially in desmoplastic areas.^10^ Thrombospondin expression and microvessel counts in schirrous carcinoma are significantly increased in comparison with those observed in usual DC.^10^ Iochim et al. have shown a positive correlation between the expression of matrix metalloproteinase with overexpression of p53 protein.^11^ Fibroblasts are able to secrete a large variety of molecules with agonistic and antagonistic effects in tumor cells, in which the imbalance between this substances could determine the neoplastic behavior.^2^ The finding of a different expression profile for p53 protein related to the amount of desmoplastic reaction could be another important difference in DC. The relationship between fibrosis and expression of p53 protein has been shown by other authors in different tissues and diseases, as idiopathic pulmonary fibrosis,^12^ squamous cell carcinoma of the vulva,^13^ and experimental development of adenocarcinoma of the pancreas.^14^

The use of desmoplastic reaction in DC as a prognostic factor has been debated in the literature. Schirrous carcinomas are usually less delimited, with less inflammatory response, and moderately differentiated.^3^ There are several papers showing that desmoplastic reaction has some importance as an isolated prognostic factor. More recently, fibrotic foci has been correlated with worse prognosis.^4-6^ Mezi et al. have demonstrated that tumors with intense desmoplastic reaction have shorter intervals free of disease, when comparing patients at similar clinical stages.^15^ This work did not address questions about survival and other clinical indicators of biological behavior. However, an important issue is to discriminate between sub-groups in schirrous carcinomas. Horiguchi et al. have demonstrated that there are no differences in expression of c-erbB-2 oncoprotein between normal DC and schirrous carcinoma, but the expression of c-erbB-2 oncoprotein is a independent prognostic factor in schirrous carcinoma.^16^ We now intend to test the importance of the different biological markers, with emphasis on p53 protein, as prognostic factors in schirrous carcinoma.

## CONCLUSION

Infiltrating ductal carcinomas of the breast with intense desmoplastic reaction (schirrous carcinoma) present overexpression of p53 protein in comparison with other DC with less prominent desmoplastic reaction. There are no differences between the intensity of the desmo-plastic reaction and immunohistochemical expression of estrogen and progesterone receptors, and with c-erbB-2 oncoprotein.

Further studies are necessary to determine whether the overexpression of p53 protein in schirrous carcinoma is a primary phenomenon characterized by mutation of the p53 gene, or secondary to the interactions with other molecules embedded in the fibrous tumor stroma.
